# Supplementary material for: Protein nanowires with tunable functionality and programmable self-assembly using sequence-controlled synthesis
Source: Nat Commun. 2022 Feb 11;13:829. doi: 10.1038/s41467-022-28206-x (PMC8837800; doi:10.1038/s41467-022-28206-x)
Supplement: Supplementary file 1 — Supplementary information [file 41467_2022_28206_MOESM1_ESM.pdf]

Supplementary Information Inventory

Protein nanowires with tunable functionality and programmable self-assembly using sequence-controlled synthesis

Daniel Mark Shapiro[1,2,3,4], Gunasheil Mandava[3,4], Sibel Ebru Yalcin[3,4], Pol Arranz-Gibert[1,2], Peter Dahl[3,4], Catharine Shipps[3,4], Yangqi Gu[3,4], Vishok Srikanth[3,4], Aldo I. Salazar Morales[3,4], J. Patrick O'Brien[3,4], Koen Vanderschuren[1,2], Dennis Vu[3,4], Victor Batista[5], Nikhil Malvankar[3,4*†], Farren J. Isaacs[1,2,6*†]

[1]Department of Molecular, Cellular & Developmental Biology, Yale University, New Haven, CT, 06520, USA.
[2]Systems Biology Institute, Yale University, West Haven, CT, 06516, USA.
[3]Department of Molecular Biophysics and Biochemistry, Yale University, New Haven, CT, 06520, USA.
[4]Microbial Sciences Institute, Yale University, West Haven, CT, 06516, USA.
[5]Department of Chemistry, Yale University, New Haven, CT 06520, USA
[6]Department of Biomedical Engineering, Yale University, New Haven, CT, 06520, USA.

[†]Equal contribution
[*]Correspondence to: Email: nikhil.malvankar@yale.edu (N.S.M.); farren.isaacs@yale.edu (F.J.I.)

Contents:

1. Supplementary Data
    a. Supplementary Figure 1……………………………………2
    b. Supplementary Figure 2……………………………………3
    c. Supplementary Figure 3……………………………………4
    d. Supplementary Figure 4……………………………………5
    e. Supplementary Figure 5……………………………………6
    f. Supplementary Figure 6……………………………………7
    g. Supplementary Figure 7……………………………………8
    h. Supplementary Table 1……………………………………9
    i. Supplementary Table 2……………………………………9
    j. Supplementary Table 3……………………………………10
    k. Supplementary References………………………………..12
2. Source Data File in .xlsx format (separate)
3. MD Analysis Tcl script in .tcl format (separate)

Supplementary Data

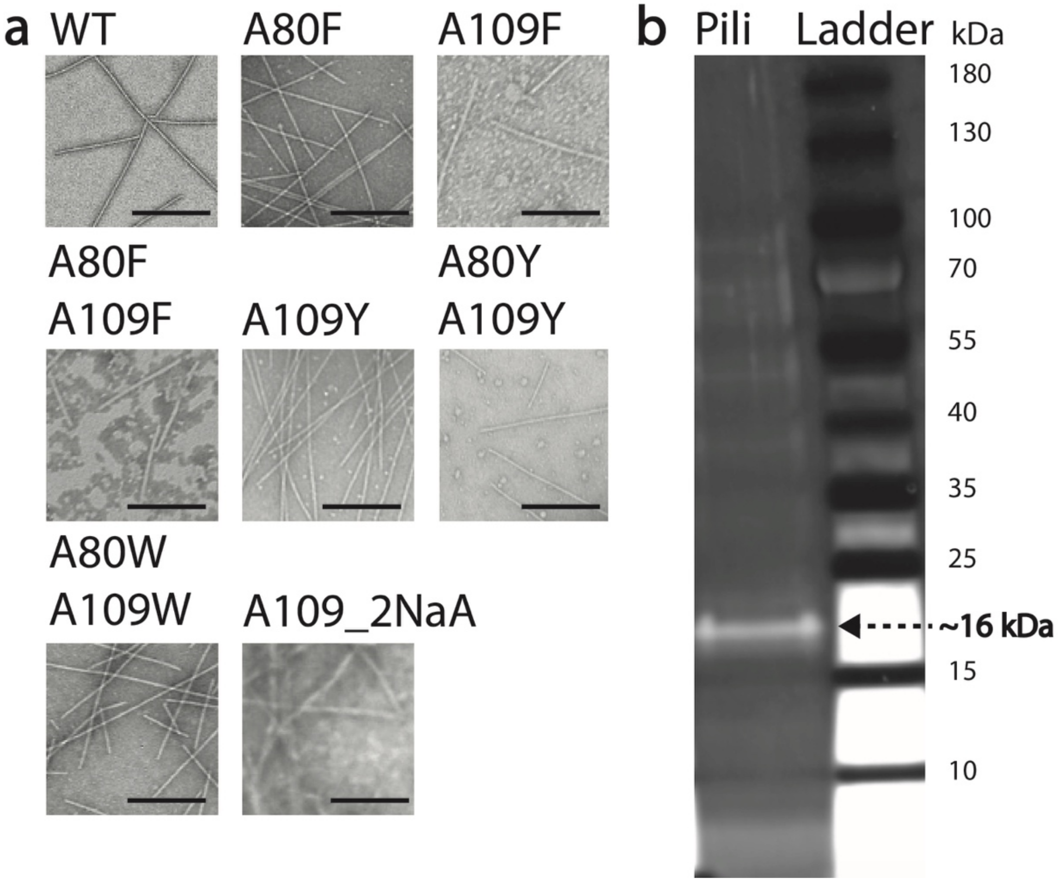

Supplementary Fig. 1: TEM of Pili filaments, SDS-PAGE gel. a) TEM images of pili show consistent pili structure and morphology. Scale bar: 200 nm. b) A SYPRO Ruby-stained SDS-PAGE gel showing the pure FimA A80W A109W band of the sample. The predicted molecular weight of mature, depolymerized FimA A80W A109W based on the translated sequence is 16.058 kDa. Note that as there is no other contaminating protein present this gel demonstrates the efficacy of the purification method in this method in producing pure homogenous nanowire samples. This purification was conducted once on a representative sample.

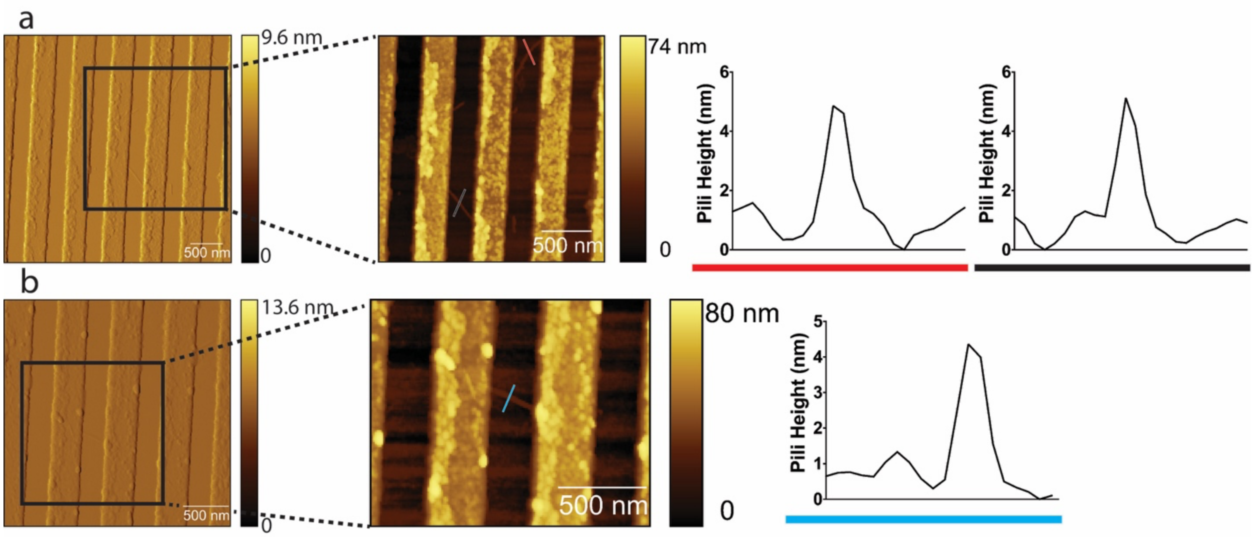

Supplementary Fig. 2: AFM images and height profiles of pili crossing electrodes. Atomic force microscopy (AFM) images of pili crossing electrodes. a,b) representative images and height profiles of pili crossing electrodes after being drop cast onto the device. Height profiles measured across the diameter of the pili show height consistent with pilus structure. Colored bars correspond to height profiles.

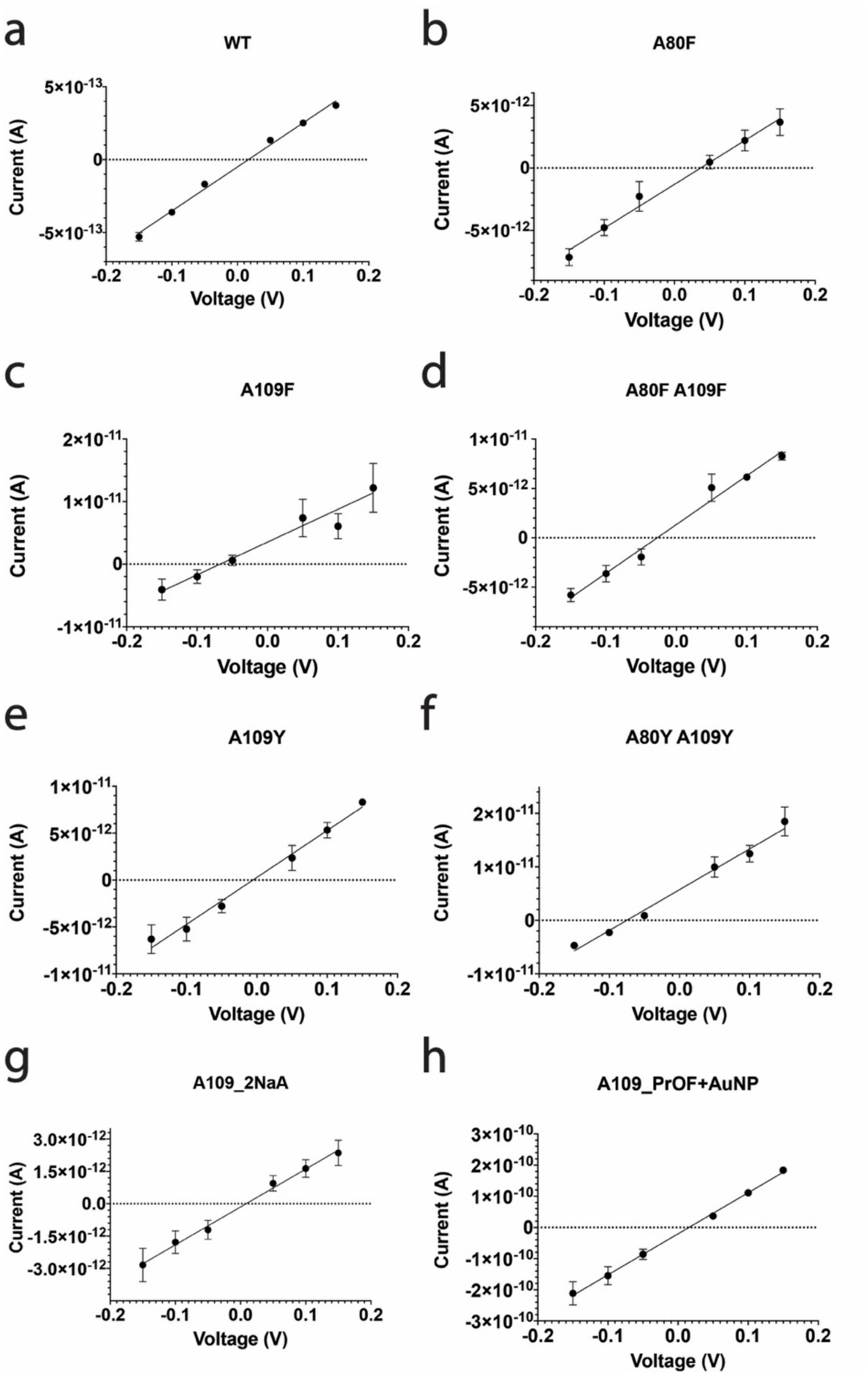

Supplementary Fig. 3: I-V curves for conductivity measurements. Current-Voltage (IV) measurements for all pili variants for which conductivity was measured. IV measurements of pili show linear current dependence on voltage for small, physiologically relevant voltages. Each data point is for n=3 at that voltage. Error bars represent s.e.m.

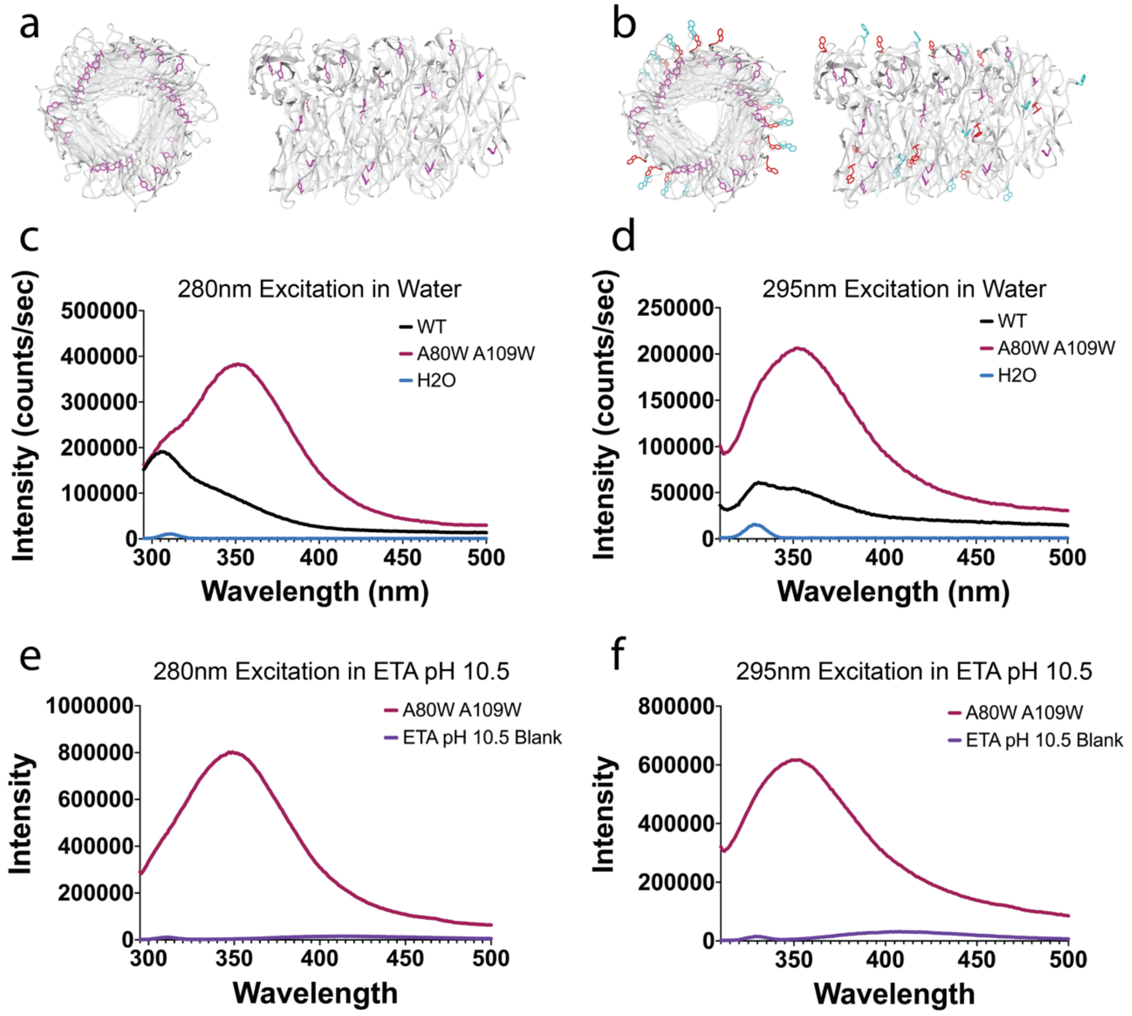

Supplementary Fig. 4: Fluorescence measurements on FimA A80W A109W pili confirm tryptophan residues are solvent-exposed, indicating no significant conformational change. a) The structure of a section of *E. coli* type 1 pili consisting of eight WT FimA protein monomers with tyrosine residues colored in purple. Note that there are no tryptophan residues in WT pili. b) A section of *E. coli* type 1 pili consisting of eight FimA A80W A109W protein monomers, with the tryptophan residues colored in cyan (position 80) and red (position 109) and tyrosine residues colored in purple. c,d) Exciting the protein at 280 nm in water at pH 7 matches the λmax of tryptophan but also excites tyrosine[1] while exciting the protein at 295 nm in water at pH 7 ensures that only tryptophan is excited. c) The fluorescence spectrum excited at 280 nm, with the black line representing pili made of WT FimA and the purple line representing pili made of FimA A80W A109W. The tyrosine residues are responsible for the peak at 306 nm from the 280 nm excitation[1]. d) The fluorescence spectrum excited at 295 nm, with the black line representing pili made of WT FimA and the purple line representing pili made of FimA A80W A109W. The emission peak around 352 nm for both excitation wavelengths confirms that the incorporated tryptophan residues are solvent-exposed. e,f) Exciting the protein at 280 or 295 nm in ETA pH 10.5 shows the same tryptophan peak, demonstrating that tryptophan remains solvent exposed and there is no major structural change in ETA pH 10.5.

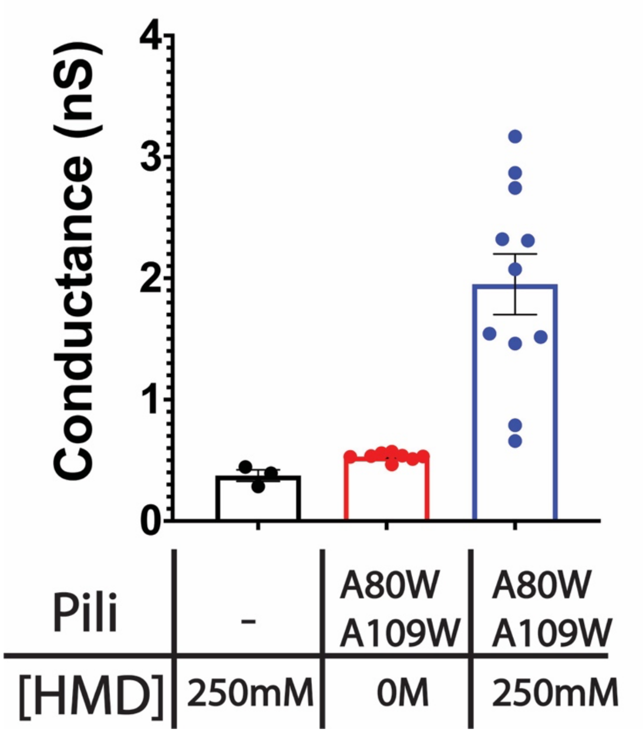

Supplementary Fig. 5: Conductance of pili nanostructures. Conductance of HMD only shows a high baseline conductance of 0.3737 ± .0472 nS, however conductance of unordered networks of FimA A80W A109W pili remains higher than background HMD at 0.5297 ± 0.0113 nS. The conductance of bundled filaments is still significantly higher at 1.950 ± 0.2495 nS. A Student's t-test was performed between ordered (A80W A109W pili, 250 mM HMD) and unordered (A80W A109W pili, 0 mM HMD), and the p-value = 0.00007. It was also done between ordered and the control (250 mM HMD) and the p-value = 0.0002. All experiments were performed independently, and error bars represent s.e.m. HMD only: n = 3. Unordered pili: n = 8. Bundled pili: n = 11.

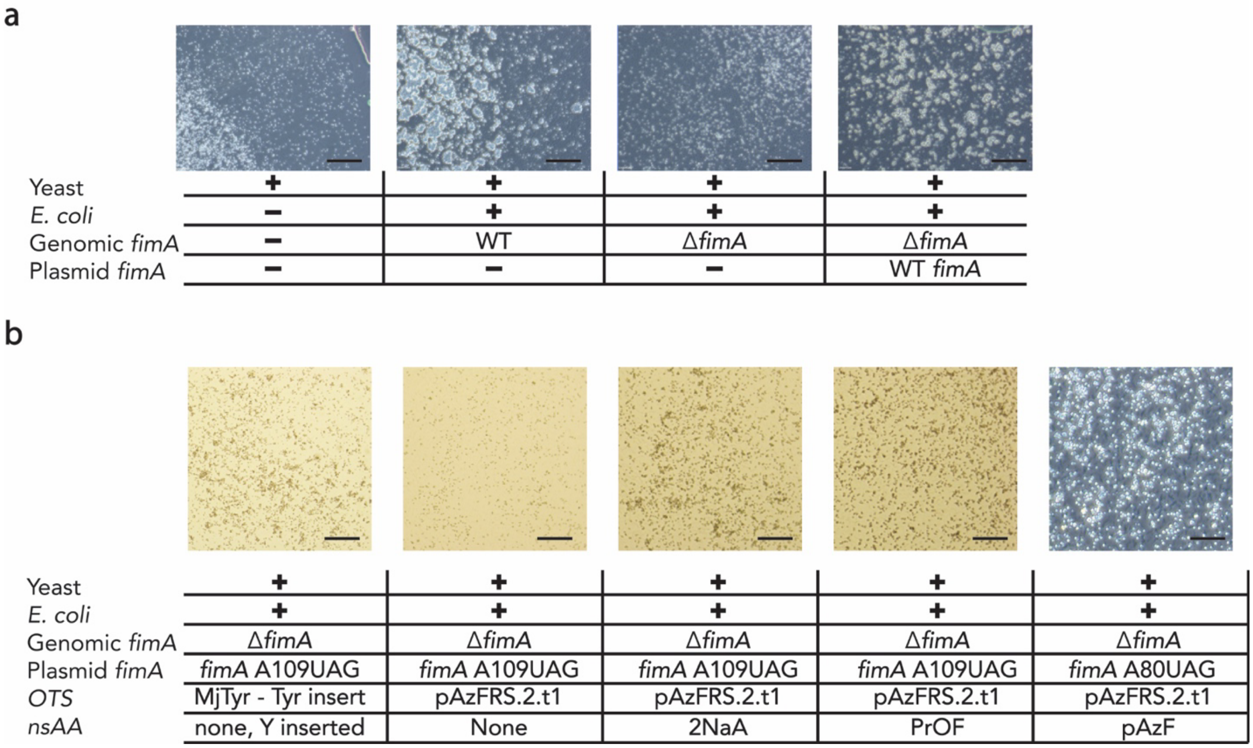

Supplementary Fig. 6: Yeast agglutination assays for *E. coli* pili production confirm pili expression from plasmid and incorporation of tyrosine, 2NaA, and PrOF at FimA UAG109 using an orthogonal translation system. Deletion of *fimA* from chromosome or lack of nsAA inhibits pili production and prevents agglutination phenotype. Scale bar: 200μm. a) Yeast agglutination assay images of *E. coli* mixed with yeast to demonstrate agglutination phenotype is dependent on pili expression. In images with blue background, white dots and clumps are yeast. In images with yellow background, dark brown dots and clumps are yeast. *E. coli* is not visible. Yeast agglutination behavior is dependent on *E. coli* pili expression, either from the genome or from a plasmid as used in this study. Deleting *fimA* from the genome abolishes yeast agglutination, while expressing *fimA* from a plasmid rescues yeast agglutination. Experiment repeated greater than 10 times with similar results. b) Yeast agglutination behavior arising from incorporation of an amino acid at the FimA UAG109 position that allows for pili expression. All pili proteins are expressed from a plasmid. The MjTyr OTS (leftmost panel) inserts tyrosine at UAG codons, creating successfully assembled FimA A109Y pili which lead to agglutination. When no nsAA is supplemented, pili are not expressed, and no agglutination is seen (2nd image from left). Adding 2NaA and PrOF leads to yeast agglutination when incorporated at position FimA 109 and adding pAzF leads to yeast agglutination when incorporated at position FimA 80. nsAA experiments independently repeated twice.

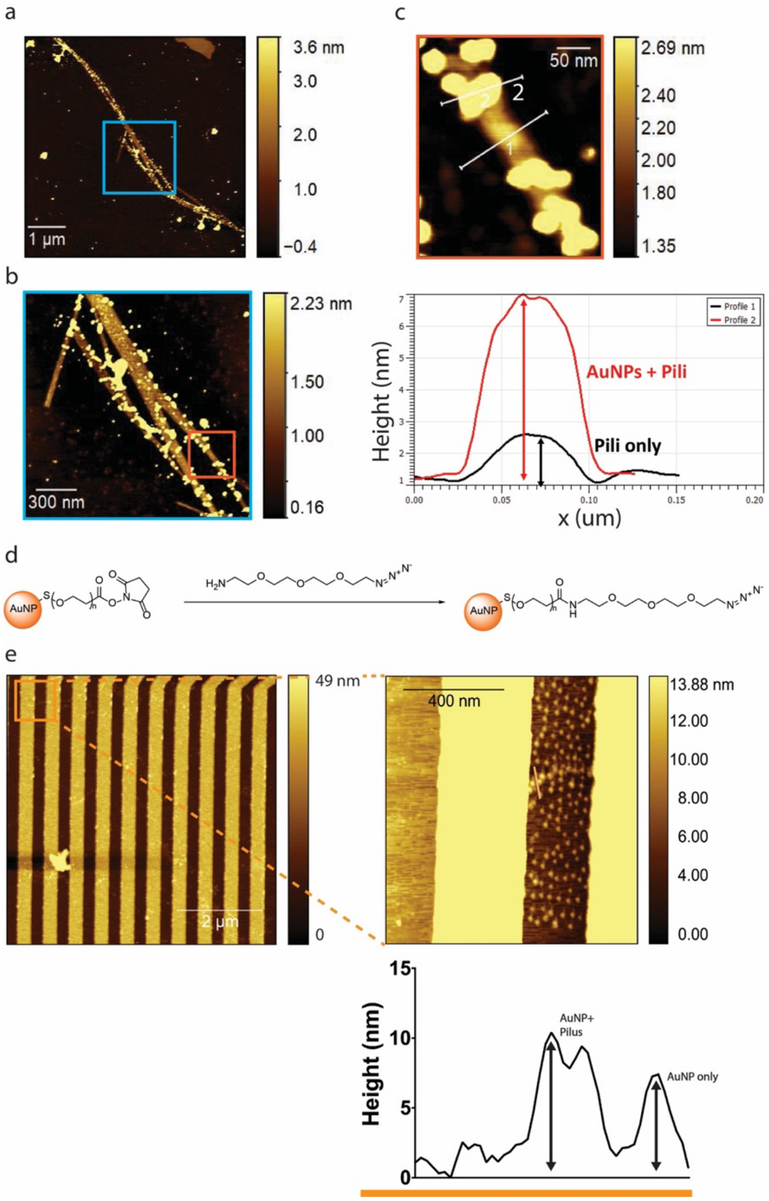

Supplementary Fig. 7: AuNP-decorated Pili. AFM Images of AuNP-decorated pili demonstrate consistent coverage of Type 1 pili with incorporated PrOF with azide-functionalized AuNPs after Cu-catalyzed click reaction. a) Representative AuNP-decorated pili. Blue box is zoomed in view in b. b) Zoomed in AuNP-decorated pili, orange box is zoomed in view in c. c) Image of single AuNP-decorated pilus with associated height profiles. Pili imaged after Cu-catalyzed click reaction are consistently 2nm in diameter, purchased AuNPs are 5nm in diameter. Cross-section of AuNPs attached to pilus is 7 nm, indicating AuNP with pilus protein underneath. For parts a-c, experiments were repeated independently 3 times with similar results. d) 5 nm N Hydroxysuccinimide (NHS)-functionalized AuNPs were purchased from Cytodiagnostics. To change the terminal NHS group to an azide group, the NHS group was covalently bound to the amine group of the 11-Azido-3,6,9-trioxaundecan-1-amine azide linker (methods). e) Representative AFM image of AuNP-decorated pilus crossing two electrodes. Height profile corresponds to height across diameter of pilus+AuNPs. As seen from this image, in all measured cases AuNPs decorated entire pilus between electrodes.

Supplementary Table 1: Standard Amino Acid mutations made in type 1 pili

Mutation in *fimA*	Pili Expression	σ (mS/cm)
WT	Yes	0.5198 ± 0.1475
A80F	Yes	5.168 ± 0.2660
A109F	Yes	5.933 ± 0.5713
A80F A109F	Yes	7.250 ± 1.355
A80F H82F A109F	No	
A80F H82F A109Y	No	
A80Y	Yes	9.208 ± 0.6528
A109Y	Yes	Not Measured
A80Y A109Y	Yes	9.287 ± 0.872
A80W	Yes	Not Measured
A109W	Yes	Not Measured
A80W A109W	Yes	43.48 ± 4.572
A80Y H82F A80Y	No	
A80Y H82Y A109Y	No	

Supplementary Table 2: nsAAs inserted, position, and conductivity

Position and nsAA	Pili expression	σ (mS/cm)
High Aromaticity		
A80 2NaA (3-(2-Naphthyl)-L-alanine)	No	
A109 2NaA	Yes	2.709 ± 0.1674
A80 PhF (4-phenyl-phenylalanine)	No	
A109 PhF	No	
A109 4ClF (4-chloro-phenylalanine)	No	
A109 4BrF (4-bromo-phenylalanine)	No	
A109 4IF (4-bromo-phenylalanine)	No	
A109 pAcF (para-acetyl-L-phenylalanine)	No	
A109 pAF (para-amino-L-phenylalanine)	No	
Conjugation Scaffold		
A80 PrOF (4-propargyloxy-L-phenylalanine)	No	
A109 PrOF-AuNP	Yes	87.39 ± 8.607
A80 pAzF (para-azido-L-phenylalanine)	Yes	Not Measured
A109 pAzF	No	

Supplementary Table 3: Relevant DNA sequences used in this study

WT fimA sequence of *E. coli* C321.A.

The mutation locations are based on the sequence without the signal peptide (highlighted in yellow). Locations 80 and 109 are highlighted in bold blue and bold red, respectively.

atgAAAATTAAAACTCTGGCAATCGTTGTTCTGTCGGCTCTGTCCCTCAGTTCTACAGCGGCTC
TGGCCGCTGCCACGACGGTTAATGGTGGGACCGTTCACTTTAAAGGGGAAGTTGTTAACGCCGC
TTGCGCAGTTGATGCAGGCTCTGTTGATCAAACCGTTCAGTTAGGACAGGTTCGTACCGCATCG
CTGGCACAGGAAGGAGCAACCAGTTCTGCTGTCGGTTTTAACATTCAGCTGAATGATTGCGATA
CCAATGTTGCATCTAAAGCCGCTGTTGCCTTTTTAGGTACGGCGATTGATGCGGGT**CAT**ACCAA
CGTTCTGGCTCTGCAGAGTTCAGCTGCGGGTAGCGCAACAAACGTTGGTGTGCAGATCCTGGAC
AGAACGGGT**GCT**GCGCTGACGCTGGATGGTGCGACATTTAGTTCAGAAACAACCCTGAATAACG
GAACCAATACCATTCCGTTCCAGGCGCGTTATTTTGCAACCGGGGCCGCAACCCCGGGTGCTGC
TAATGCGGATGCGACCTTCAAGGTTCAGTATCAATAA

MAGE oligonucleotides

A table of all oligonucleotides used to create chromosomal mutations in the *fimA* gene. The mutations are highlighted in **bold**. An asterisk * denotes a phosphorothioate bond.

Mutation in fimA	Oligonucleotide sequence with mutation highlighted.
A109F	A*T*TCAGGGTTGTTTCTGAACTAAATGTCGCACCATCCAGCGTCAGCGC**GAA**ACCCGTTCTGTCCAGGATCTGCACACCAACGTTTGTTGC
A80F	A*G*CTGAACTCTGCAGAGCCAGAACGTTGGTATGACC**GAA**ATCAATCGCCGTACCTAAAAAGGCAACAGCGGCTTTAGATGCAACATTGGT
H82F	A*G*CTGAACTCTGCAGAGCCAGAACGTTGGT**GAA**ACCCGCATCAATCGCCGTACCTAAAAAGGCAACAGCGGCTTTAGATGCAACATTGGT
A109Y	A*T*TCAGGGTTGTTTCTGAACTAAATGTCGCACCATCCAGCGTCAGCGC**ATA**ACCCGTTCTGTCCAGGATCTGCACACCAACGTTTGTTGC
A80Y	A*G*CTGAACTCTGCAGAGCCAGAACGTTGGTATGACC**ATA**ATCAATCGCCGTACCTAAAAAGGCAACAGCGGCTTTAGATGCAACATTGGT
H82Y	A*G*CTGAACTCTGCAGAGCCAGAACGTTGGT**ATA**ACCCGCATCAATCGCCGTACCTAAAAAGGCAACAGCGGCTTTAGATGCAACATTGGT
A80F H82F	A*G*CTGAACTCTGCAGAGCCAGAACGTTGGT**GAA**ACC**GAA**ATCAATCGCCGTACCTAAAAAGGCAACAGCGGCTTTAGATGCAACATTGGT
A80Y H82Y	A*G*CTGAACTCTGCAGAGCCAGAACGTTGGT**ATA**ACC**ATA**ATCAATCGCCGTACCTAAAAAGGCAACAGCGGCTTTAGATGCAACATTGGT
A80F H82Y	A*G*CTGAACTCTGCAGAGCCAGAACGTTGGT**GAA**ACC**ATA**ATCAATCGCCGTACCTAAAAAGGCAACAGCGGCTTTAGATGCAACATTGGT
A80Y H82F	A*G*CTGAACTCTGCAGAGCCAGAACGTTGGT**ATA**ACC**GAA**ATCAATCGCCGTACCTAAAAAGGCAACAGCGGCTTTAGATGCAACATTGGT
A109W	A*T*TCAGGGTTGTTTCTGAACTAAATGTCGCACCATCCAGCGTCAGCGC**CCA**ACCCGTTCTGTCCAGGATCTGCACACCAACGTTTGTTGC
A80W	A*C*CCGCAGCTGAACTCTGCAGAGCCAGAACGTTGGTATGACC**CCA**ATCAATCGCCGTACCTAAAAAGGCAACAGCGGCTTTAGATGCAAC
A80F A84F	G*C*GCTACCCGCAGCTGAACTCTGCAGAGCCAGAAC**GAA**GGTATGACC**GAA**ATCAATCGCCGTACCTAAAAAGGCAACAGCGGCTTTAGAT
A80Y A84Y	A*G*CTGAACTCTGCAGAGCCAGAAC**ATA**GGTATGACC**ATA**ATCAATCGCCGTACCTAAAAAGGCAACAGCGGCTTTAGATGCAACATTGGT
A84F	G*C*GCTACCCGCAGCTGAACTCTGCAGAGCCAGAAC**GAA**GGTATGACCCGCATCAATCGCCGTACCTAAAAAGGCAACAGCGGCTTTAGAT
A84Y	G*C*GCTACCCGCAGCTGAACTCTGCAGAGCCAGAAC**ATA**GGTATGACCCGCATCAATCGCCGTACCTAAAAAGGCAACAGCGGCTTTAGAT

Site-directed mutagenesis primers used in this study:
Mutations FimA A80TAG and A109TAG were created using site-directed mutagenesis on the pSHDS.1 plasmid as in *methods*. Forward (F) and reverse (R) primers are listed below for each mutation.

fimA A80TAG F	gggtCATACCAACGTTCTGGCTC
fimA A80TAG R	taatcAATCGCCGTACCTAAAAAG
fimA A109TAG F	ggcgCTGACGCTGGATGGTGCG
fimA A109TAG R	taaccCGTTCTGTCCAGGATCTGC

References

1 Pain, R. H. Determining the Fluorescence Spectrum of a Protein. *Current Protocols in Protein Science* **38**, 7.7.1-7.7.20, doi:https://doi.org/10.1002/0471140864.ps0707s38 (2004).
