# Supplementary material for: Protein nanowires with tunable functionality and programmable self-assembly using sequence-controlled synthesis
Source: Nat Commun. 2022 Feb 11;13:829. doi: 10.1038/s41467-022-28206-x (PMC8837800; doi:10.1038/s41467-022-28206-x)
Supplement: Supplementary file 4 — Description of Additional Supplementary Files [file 41467_2022_28206_MOESM4_ESM.pdf]

Title: Supplementary Software

Description: First, the code imports the files containing the (x,y,z) coordinates of all atoms of the two FimA A80W A109W monomers and the HMD molecules over the course of the simulation. From then on, for each timepoint, the code calculates the distance between the geometric centers of the FimA A80W A109W monomers as follows. The code calculates the geometric center (x,y,z) point of each monomer as the minimum x, y, or z atom location within the monomer plus 1/2 the distance between the minimum and maximum x, y, or z atom location within the monomer in each dimension (e.g. center_x = min(x) + (max(x)-min(x))/2). It then calculates the Euclidian distance between the geometric centers of the two monomers (e.g. distance = sqrt((center_x_monomer2-center_x_monomer1)^2 + (center_y_monomer2-center_y_monomer1)^2 + (center_z_monomer2-mid_z_monomer1)^2)). The Euclidian distance between the two monomers is thus recorded at each timepoint and presented in Figure 3b.
